# Pathogenic Mycobacterium bovis strains differ in their ability to modulate the proinflammatory activation phenotype of macrophages

**DOI:** 10.1186/1471-2180-12-166

**Published:** 2012-08-03

**Authors:** Marcelle RM Andrade, Eduardo P Amaral, Simone CM Ribeiro, Fabricio M Almeida, Tanara V Peres, Verônica Lanes, Maria Regina D’Império-Lima, Elena B Lasunskaia

**Affiliations:** 1 Laboratory of Biology of Recognition, Universidade Estadual do Norte Fluminense, Campos Rio de Janeiro 28013-602, Brazil; 2 Department of Immunology, Universidade de São Paulo, São Paulo, 05508-900, Brazil

**Keywords:** Bone marrow- derived macrophages, Mycobacterium bovis, Macrophage activation phenotype, Interferon-γ, IL-10

## Abstract

**Background:**

Tuberculosis, caused by *Mycobacterium tuberculosis* or *Mycobacterium bovis,* remains one of the leading infectious diseases worldwide. The ability of mycobacteria to rapidly grow in host macrophages is a factor contributing to enhanced virulence of the bacteria and disease progression. Bactericidal functions of phagocytes are strictly dependent on activation status of these cells, regulated by the infecting agent and cytokines. Pathogenic mycobacteria can survive the hostile environment of the phagosome through interference with activation of bactericidal responses. To study the mechanisms employed by highly virulent mycobacteria to promote their intracellular survival, we investigated modulating effects of two pathogenic *M. bovis* isolates and a reference *M. tuberculosis* H37Rv strain, differing in their ability to multiply in macrophages, on activation phenotypes of the cells primed with major cytokines regulating proinflammatory macrophage activity.

**Results:**

Bone marrow- derived macrophages obtained from C57BL/6 mice were infected by mycobacteria after a period of cell incubation with or without treatment with IFN-γ, inducing proinflammatory type-1 macrophages (M1), or IL-10, inducing anti-inflammatory type-2 cells (M2). Phenotypic profiling of M1 and M2 was then evaluated. The *M. bovis* strain MP287/03 was able to grow more efficiently in the untreated macrophages, compared with the strains B2 or H37Rv. This strain induced weaker secretion of proinflammatory cytokines, coinciding with higher expression of M2 cell markers, mannose receptor (MR) and arginase-1 (Arg-1). Treatment of macrophages with IFN-γ and infection by the strains B2 and H37Rv synergistically induced M1 polarization, leading to high levels of inducible nitric oxide synthase (iNOS) expression, and reduced expression of the Arg-1. In contrast, the cells infected with the strain MP287/03 expressed high levels of Arg-1 which competed with iNOS for the common substrate arginine, leading to lower levels of NO production.

**Conclusions:**

The data obtained demonstrated that the strain, characterized by increased growth in macrophages, down- modulated classical macrophage activation, through induction of an atypical mixed M1/M2 phenotype.

## Background

Macrophages are key cells of innate immunity to different mycobacterial infections, including human and bovine tuberculosis caused predominantly by *Mycobacterium tuberculosis* (Mtb) and *Mycobacterium bovis* (Mbv), respectively. The functions of MΦ after infection with mycobacteria are strictly dependent on the activation phenotype, which is determined by bacteria- induced signaling through the pattern-recognition receptors (PRRs), leading to innate MΦ activation, and is also regulated by a variety of signals from the surrounding microenvironment. The most important of these signals are cytokines produced by activated lymphocytes and other cells.

Macrophages primed with Th1 cytokine (IFN-γ) polarize to proinflammatory M1 cells, readily increasing the level of activation in the presence of microbial ligands, and developing the phenotype typical of classically activated macrophages, CAM [1]. These cells produce large amounts of proinflammatory cytokines and generate reactive oxygen (ROI) and nitrogen (RNI) intermediates which enhance bactericidal and cytotoxic MΦ functions. In contrast, macrophages activated with Th2 cytokines (IL-4, IL-13), exposed to immune complexes in combination with TLR ligands, or IL-10, polarize to distinct M2 phenotypes, M2a, M2b and M2c, respectively, associated with alternatively activated macrophages (AAM), which display anti-inflammatory and tissue repair activities [2]. The M2 macrophages are characterized by expression of typical markers, including increased arginase 1 (Arg-1) expression and activity, increased expression of scavenger and mannose (MR/CD206) receptors, and production of the anti-inflammatory cytokine (IL-10), which is more pronounced in the AAM induced by exogenic IL-10. The MΦ primed by IL-10 were demonstrated to secrete none, or very low levels, of proinflammatory cytokines in response to bacterial LPS, but produce anti-inflammatory IL-10 and TGF-β, that prompted Gordon to term this M2 state the ‘innate/acquired inactivation’ [1] and include these cells to group of ‘regulatory’ MF [3]. The cytokines produced by Th1 and Th2 cells or IL-10 induce extreme phenotypes of MΦ activation characterized by distinct markers. After infection, microbial products can modulate MΦ activation through PRR-dependent signaling, providing a wide range of MΦ phenotypes between the two extremes [4].

During acute inflammatory responses to Mtb, macrophages are typically polarized to M1 under the effects of mycobacterial agonists for PRRs and IFN-γ produced by Th1, and exert potent anti-microbial effects [5]. The transcriptomic analysis of responses of murine bone marrow- derived macrophages (BMDM) to Mtb and IFN-γ revealed an overlap of genes modulated by mycobacteria and IFN − γ, which corresponded to a M1 profile [6,7]. In contrast, pretreatment of the BMDM with IL-4 resulted in the M2 transcriptional profile, and these cells presented delayed, and partially diminished, anti-mycobacterial responses [7]. These data were obtained employing the ‘laboratory’ Mtb strain H37Rv, widely used as a reference virulent strain for studies of tuberculosis pathogenesis. However, there is mounting evidence that strains of Mtb and Mbv circulating in human and animal populations are more genetically and functionally diverse than previously appreciated, demonstrating strain-dependent variation in virulence [8-11]. In the model of MΦ infection, highly virulent and epidemiologically successful strains of Mtb were able to grow faster than the less virulent isolates [12,13]. The enhanced bacterial growth was observed not only in the intact murine MΦ, but also in those primed by IFN-γ [14,15], suggesting, that at least some virulent strains of Mtb were able to inhibit CAM. Additionally, highly virulent Mtb were able to switch the initial Th1-type reaction, associated with high levels of IFN-γ production in the infected mice, to potent Treg cell response leading to production of IL-10, which reduced the bactericidal activities of MΦ [11]. In contrast to Mtb, modulating effects of pathogenic Mbv strains, differing in virulence-associated properties, on the MΦ activation phenotypes, determined by main regulating cytokines, IFN-γ and IL-10, have not been yet elucidated.

In this work, we studied the effects of pathogenic Mbv isolates and reference Mtb strain H37Rv, differing in their ability to grow intracellularly in murine MΦ, on polarization of these cells to M1 and M2 phenotypes induced by the treatment with IFN-γ and IL-10, respectively. Expression levels of typical M1 and M2 markers were evaluated. Additionally, we verified intracellular signaling pathways that could regulate production of microbicidal RNIs, through the modulation of iNOS and Arg-1 expression.

Our results demonstrated that the Mbv strain MP287/03, characterized by increased intracellular survival and growth, in contrast to other strains, inhibited classical MΦ activation, switching the M1 activation profile of the cells, stimulated with IFN-γ, to a mixed M1/M2 phenotype. Increased expression of Arg-1, observed in these cells, coincided with low levels of nitric oxide production, suggesting that reduced exposure of bacteria to nitrosative stress contributed to increased intracellular survival of these bacteria.

## Results

### Pathogenic isolates of *M. bovis* differed in their capacity to grow in the cultured macrophages

**Figure 1:**
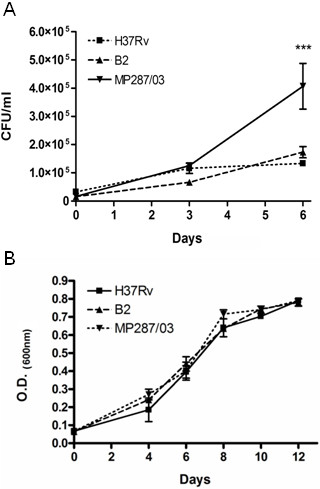
**Evaluation of the growth properties of *M. bovis *isolates.** Isolates obtained from animals with tuberculosis, strains MP287/03 and B2, and reference *M. tuberculosis* strain H37Rv, were used for infection of BMDM in vitro (**A**) or cultured in Middlebrook 7H9 broth (**B**). Growth rates of mycobacteria inside MΦ infected at MOI of 1 were determined using the colony count method. Intracellular CFU numbers were quantified immediately after infection (day 0) or at 3 or 6 days after infection (**A**). Growth rates of mycobacteria in 7 H9 Middlebrook broth were monitored by measurement of OD of the mycobacterial cultures by spectrophotometry. The growth curves of the mycobacterial strains within a 12 day period of incubation are presented. (**B**). Values are the means ± SD of three independent experiments with samples in triplicate.

To investigate the mechanisms employed by pathogenic Mbv to modulate MΦ activation, we selected for this study two clinical isolates of Mbv which showed significant difference in capacity of bacteria to grow in MΦ. As shown in Figure 1A, growth kinetics of one of the Mbv isolates, strain B2, was similar to that of the reference Mtb strain H37Rv. In contrast, the Mbv strain MP287/03 grew in MΦ significantly faster (p < 0.001). After six days of incubation, an increase in the numbers of intracellular bacteria was 3-fold higher in cultures infected by the strain MP287/03, than those infected by strain B2. In contrast to the intracellular growth, growth rate of the tested strains in specific Middlebrook 7H9 media was similar, demonstrating that the intrinsic abilities of the different strains to replicate were similar (Figure 1B). These data suggested that the observed differences in intracellular growth of these bacteria could be associated with differential resistance of the bacterial strains to microbicidal effects of MΦ.

**Figure 2:**
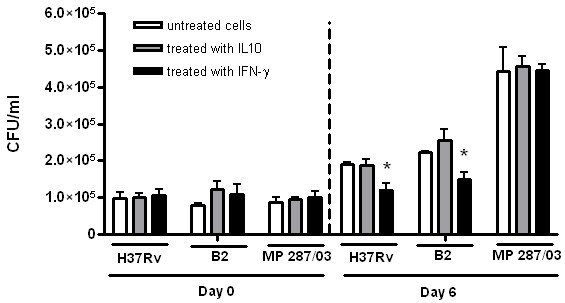
**The capacity of pathogenic mycobacteria to grow intracellularly in macrophages treated with IFN-γor IL-10.** Cultures of BMDM were pretreated with exogenic murine r-IFN -γ or r-IL-10 for 2 h, infected with the mycobacterial strains at a MOI of 1, as indicated in the legend to Figure 1, and incubated in the presence of these cytokines for an additional 6 days. The intracellular CFU numbers determined at day 0 and day 6 are presented. The data of three independent experiments are shown as mean ± SD of samples in triplicate. Asterisks represent statistical significance (p < 0.05) compared to infected cells cultured without addition of the cytokines.

The main cytokines regulating proinflammatory MΦ activity, IFN-γ [16] and IL-10 [17], are known to increase or decrease the bactericidal functions of these cells, respectively. To verify whether intracellular survival of the different mycobacterial strains are equally regulated by the effects of IFN-γ and IL-10 on MΦ, we tested intracellular growth rates of the studied bacterial strains in BMDM cultured in the presence of these cytokines. As shown in Figure 2, the treatment of macrophage cultures with recombinant IL-10 had no significant effect on the growth of the studied strains. Treatment with IFN-γ significantly reduced the growth rate of the strains B2 and H37Rv, but this effect was less pronounced in the cell cultures infected with the strain MP287/03. These data demonstrated the resistance of rapidly growing strain (MP287/03) to the effects of IFN-γ, which is probably mediated by the ability of the mycobacteria to inhibit classic macrophage activation induced by this cytokine.

### Innate macrophage activation by the pathogenic mycobacterial strains differing in growth kinetics in macrophages

To study the effects of pathogenic Mbv isolates on MΦ activation, we evaluated characteristic markers of M1- and M2- type macrophage polarization induced in infected BMDM, in the presence or absence of IFN-γ and IL-10.

**Figure 3:**
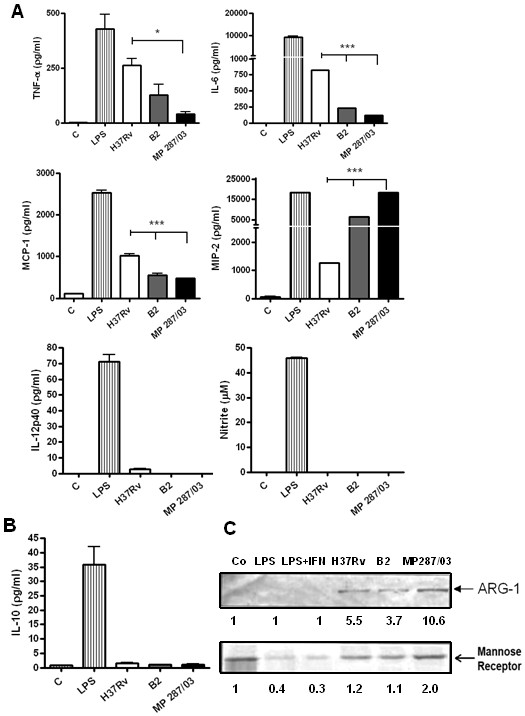
**The activation profiles of macrophages infected with pathogenic mycobacteria. BMDM were infected with the studied mycobacterial strains at a MOI of 5:1, washed and incubated for an additional 48 h.** The cells left untreated and cells stimulated with LPS for 48 h were used as a negative and positive controls of proinflammatory activation, respectively. To evaluate markers of M1-type activation (**A**), the culture supernatants of infected cultures were harvested and tested for TNF-α, IL-6, MCP-1, MIP-2 and IL-12 by Bioplex test, and for NO production by Griess reaction. Assays were completed with duplicate samples, and results are expressed as a mean of three independent experiments. Lines over bars indicate the isolates for which the induced cytokine production differed significantly from that induced by H37Rv (*p < 0.05; ***p < 0.001). To evaluate markers of M2-type activation, secretion of IL-10 was quantified by Bioplex assay (**B**), and expression of Arginase 1 and MR/CD206 in the adhered cells was tested by Western blotting (**C**). Lower panel, quantification of the protein levels by densitometric analysis of immunoreactive bands.

First, we investigated the innate MΦ activation induced by infection. Evaluation of expression of the M1 proinflammatory markers, including factors mediating recruitment of the phagocytic cells (MCP-1/CCL2 and MIP-2/CXCL2), and contributing to the MΦ microbicidity (TNF-α, IL-12, IL-6 and NO), demonstrated that the studied pathogenic mycobacterial strains induced different patterns of cytokine secretion by the BMDM (Figure 3A). Both clinical isolates of Mbv induced less IL-6 and MCP-1, and, additionally, the Mbv strain MP287/03 induced less TNF-α, than the reference strain H37Rv. In contrast, the level of secretion of MIP-2, an important chemokine regulating migration of granulocytes, was significantly increased in cultures infected with the Mbv strains. These cells secreted 10-fold more MIP-2 than the cells infected by H37Rv strain, and 3-fold more than those infected by the strain B2. Neither mycobacterial strain tested in this study was able to induce in MΦ the production of NO or IL-12, although production of these mediators was induced by the LPS (Figure 3A).

Evaluation of the expression of typical M2 markers (IL-10, Arg-1 and MR/CD206) by the infected cells demonstrated that neither strain induced production of the IL-10 (Figure 3B). In contrast, all the studied mycobacterial strains were able to induce expression of Arg-1, and the highest level was observed in the cells infected with the strain MP287/03 (Figure 3C). The expression of MR, which was constitutively high in the intact uninfected BMDM, was suppressed by treatment of the cells with LPS, or infection with the less virulent H37Rv and B2, whereas the cells infected with the strain MP287/03 continued to express high level of this receptor (Figure 3C).

These data demonstrated that the proinflammatory activation of MΦ by clinical isolates of Mbv, and particularly by the fast growing strain MP287/03, was significantly lower than that induced by the LPS or reference Mtb mycobacteria. Additionally, the strain MP287/03 induced in the MΦ a more pronounced expression of some M2 markers.

However, strong secretion of proinflammatory MIP-2 chemokine observed in cell cultures infected by the strain MP287/03 suggested that these bacteria induced in MΦ an atypical, mixed M1/M2 activation phenotype.

### Modulating effects of the pathogenic mycobacterial strains on the macrophage activation phenotypes induced by the cell treatment with IFN-γ and IL-10

**Figure 4:**
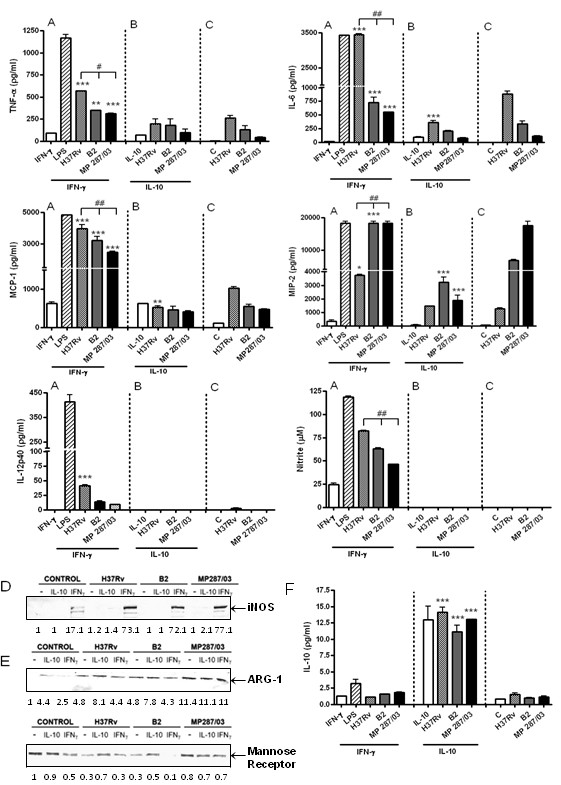
**The activation profiles of macrophages treated with IFN-**γ **or IL-10 and infected with pathogenic mycobacteria.** BMDM were pretreated, or not, with murine r-IFN-γ or r-IL-10 for 2 h, infected with the studied mycobacterial strains at a MOI of 5:1, washed, treated again with the cytokines and incubated for an additional 48 h. The cells stimulated with LPS and r-IFN-γ for 48 h, or left untreated, were used as a positive and negative controls of classical proinflammatory activation, respectively. To evaluate markers of M1-type activation, the culture supernatants were tested for proinflammatory mediator levels (**A-C**) and the adhered cells were tested for expression of iNOS (**D**). Measurement of TNF-α, IL-6, MCP-1, MIP-2 and IL-12 concentrations was performed by Bioplex test, and NO production was evaluated by Griess reaction Assays were completed with duplicate samples, and results are expressed as a mean of three independent experiments. To evaluate markers of M2-type activation, expression of Arginase 1 and MR/CD206 in the adhered cells was tested by Western blotting (**E**) and secretion of IL-10 was quantified by Bioplex assay (**F**). Lower panels in D and E, quantification of the protein levels by densitometric analysis of immunoreactive bands. Asterisks in A, B and F indicate the infected cultures treated with recombinant IFN-γ or IL-10, for which the induced cytokine production differed significantly from that in the corresponding cultures incubated without the presence of exogenic cytokines (*p < 0.05; **p < 0.01; ***p < 0.001). Lines over bars in A and B indicate the Mbv isolates for which the induced cytokine or NO production differed significantly from that induced by H37Rv (#p < 0.01; ##p < 0.001).

To study the MΦ activation phenotypes resulted from combining effects of bacteria and regulating cytokines, we evaluated expression of the markers of M1 (Figure 4A-4D) and M2 cells (Figure 4E and 4 F), by the pretreatment of infected BMDM with IFN-γ (Figure 4A), and IL-10 (Figure 4B). The markers expressed by the infected cells, which were treated with the cytokines, were compared with those of the infected cells, which were left untreated. Treatment with IFN-γ enhanced production of proinflammatory mediators in cultures infected by all the strains studied. However, the levels of secretion varied in a strain-dependent manner. Macrophages infected by the Mbv strains in the presence of IFN-γ (Figure 4A) secreted significantly less TNF-α, IL-6 and MCP-1, than those infected by the H37Rv strain. In contrast, production of MIP-2 by the cells infected with Mbv was significantly higher. As expected, treatment with IFN-γ induced in the infected MΦ, or those treated with LPS, production of NO (Figure 4A), which is an important mediator of MΦ microbicidity, tightly regulated by the IFN-γ-dependent intracellular pathways. However, the cells infected with virulent Mbv strain B2, and particularly by the strain MP287/03, secreted significantly less NO than those infected with the H37Rv strain.

To verify whether signaling pathways leading to NO production were differentially modulated by the mycobacterial strains, we evaluated induction of iNOS, the essential enzyme for the conversion of arginine to citrulline and NO. The results obtained showed that treatment with IFN-γ induced iNOS expression in the cultured macrophages, and subsequent infection of these cells with bacteria enhanced the level of enzyme expression in a similar manner (Figure 4D), demonstrating no strain-specific difference in the regulation of IFN-γ-dependent signaling which leads to transactivation of the iNOS gene.

Evaluation of expression of M2 markers in the cells pretreated with IFN-γ demonstrated suppression of Arg-1 expression induced by the strains B2 and H37Rv, but not those infected with strain MP287/03 (Figure 4E). Expression of MR by MΦ was slightly inhibited in the cell cultures treated with IFN-γ, and further reduced after infection of these cells with the strains B2 or H37Rv. In contrast, infection with the strain MP287/03 restored a high level of expression of this receptor (Figure 4E), suggesting induction of MR gene transcription due to mycobacteria in these cells. Neither bacterial strain was able to induce IL-10 in the cells pretreated with the IFN-γ (Figure 4F).

To verify the effects of mycobacterial infection on the IL-10-induced M2 polarization, the cell cultures were treated with recombinant IL-10. This treatment induced in the BMDM expression of Arg-1 (Figure 4E) and secretion of IL-10 (Figure 4F) and MCP-1 (Figure 4B). Infection of these cells with the mycobacterial strains promoted expression of M2 markers, further increasing expression of the Arg-1 and suppressing inhibition of the MR expression induced by the H37Rv and B2 strains (Figure 4E). The infected cultures continued to secrete low levels of IL-10, induced by the exogenic IL-10 pretreatment (Figure 4F). Additionally, the treatment of MΦ with IL-10 suppressed ability of some mycobacterial strains to induce increased levels of secretion of proinflammatory mediators. Significant reduction of secretion of IL-6 and MCP-1 by MΦ infected with the H37Rv strain and MIP-2 chemokine secretion, induced by the strains B2 and MP287/03, was observed (Figure 4B).

These data show that the proinflammatory activities of MΦ induced by mycobacterial infection were significantly inhibited in the cells that were infected after priming by IL-10. These cells expressed MR and increased levels of Arg-1, which were particularly high in the cells infected with MP287/03 strain. Thus, the treatment with IL-10 favored M2-type activation of the infected MΦ.

## Discussion

In this study, we aimed to investigate the modulating effects of pathogenic Mbv strains, differing in virulence-associated properties, on activation phenotypes in MΦ treated with the main cytokines regulating proinflammatory MΦ activation: IFN-γ and IL-10. Rapid growth of pathogenic mycobacteria in MΦ is one of the known factors contributing to bacterial virulence [18,19]. Therefore, for this work, we selected two Mbv isolates differing significantly in the capacity to grow in MΦ. One of these isolates, strain B2, was capable of growing in BMDM at a rate similar to that of moderately virulent Mtb strain H37Rv, whereas the intracellular multiplication of other Mbv strain (MP287/03) was significantly faster. Additionally, we demonstrated that bacteria of MP287/03 strain continued to grow rapidly in cells activated by IFN-γ, whereas the growth of the strains B2 and H37Rv was significantly inhibited under this treatment. These data suggested that the MP287/03 strain was either more resistant to the bactericidal effects of macrophages classically activated by IFN-γ, or were able to inhibit MΦ activation induced by this cytokine.

The modulating effects of the Mbv strains were evaluated in comparison to those of the reference Mtb strain H37Rv, which was demonstrated in previous studies to induce in MΦ a proinflammatory activation and synergize with IFN-γ in induction of M1-type polarization of infected cells [7,20]. In accordance with these observations, our data demonstrated that the Mtb strain H37Rv induced M1 type activation of the infected BMDM, although the level of activation was less pronounced in comparison with that induced by LPS. Activation of MΦ by the Mbv strains was even weaker than that induced by the H37Rv strain. The lowest level of proinflammatory cytokine expression was observed in MΦ infected with the fast growing Mbv strain MP287/03, although these cells produced high levels of MIP-2 chemokine. Additionally, these cells displayed increased levels of expression of M2 markers (Arg-1 and MR/CD206). Thus, the MP287/03 mycobacteria induced in MΦ an atypical, mixed M1/M2 activation phenotype that coincided with enhanced intracellular growth of the bacteria.

Most important was observation, that this strain induced weaker production of the key bactericidal factors, such as TNF-α and NO, even after pretreatment of MΦ with IFN-γ, priming these cells for M1-type activation. To study the mechanisms that could underlie the observed differences in RNI production, we looked at intracellular signaling pathways leading to NO production by the infected cells. The major regulators of NO production are iNOS and Arg -1, competitive enzymes which utilize a common substrate (L-arginine) to produce NO and citrulline, or urea and ornithine, respectively [21]. In previous study [22], induction of Arg-1 expression in MΦ by attenuated Mbv strain BCG was found to be essential for reduction of NO production, through the arginine substrate depletion mechanism, leading to promotion of the intracellular survival of these mycobacteria.

In this study, we demonstrated that pathogenic Mbv were also able to induce expression of Arg-1 in the infected MΦ. Importantly, the fast growing strain MP287/03 induced higher levels of the Arg-1, than any other studied strain, and strongly up-regulated expression of Arg-1 in IFN-γ-treated cells. Although all of the studied strains enhanced expression of iNOS, induced in cells by IFN-γ, in a similar manner, the increased level of Arg-1 observed in MΦ infected with the MP287/03 strain contributed to reduction of NO secretion by these cells. These data suggested that highly virulent Mbv, characterized by enhanced growth in MΦ could induce Arg-1 as a component of the strategy to subvert the antimicrobial activity of CAM, by hydrolyzing the substrate required for NO production.

Mechanisms leading to induction of Arg-1 expression by mycobacteria are only recently starting to be elucidated. Autocrine loop of secretion of IL-6, IL-10 and G-CSF, leading to phosphorylation of STAT3 was determined as an essential mechanism for induction of Arg-1 expression in BCG-infected MΦ [22]. However, in our study, the increased Arg-1 expression induced by the strain MP287/03, coincided with low levels of IL-6 and IL-10 secretion by the infected MΦ. These data suggested that the signaling pathways, leading to the pronounced induction of the Arg-1 by highly virulent Mbv, could differ from those induced in the BCG-infected MΦ and should be investigated further in separate study.

Another mechanism, underlying the reduced microbicidity of MΦ infected with the strain MP287/03, could be associated with capacity of these bacteria to induce in the cells expression of MR. Infection with the strain H37Rv and incubation with IFN-γ, synergistically inhibited expression of MR gene in murine BMDM [7,23], constitutively expressing high levels of MR [23], resembling in this manner, alveolar macrophages [24]. In line with these observations, infection of the cells pretreated with IFN-γ by the moderately virulent strains, H37Rv and B2, in our experiments resulted in down-regulation of MR expression. In contrast to these strains, infection of MΦ by the strain MP287/03 restored expression of MR reduced by the IFN-γ treatment. High and persistent levels of MR expression in the MΦ infected with strain MP287/03 in the presence or absence of IFN-γ suggested that these cells could be more susceptible to the deleterious effects of Mannosyl-capped lipoarabinomannan (ManLAM) expressed by the pathogenic mycobacteria. Interaction of Man-LAM with MR has been demonstrated to inhibit fusion of phagosomes with lysosomes in the infected MΦ, interfere with IFN-γ-mediated signaling in MΦ activation, as well as suppress TLR-dependent induction of expression of IL-12 and other proinflammatory cytokines [25,26]. In line with this suggestion, the infected cells expressing higher levels of MR in our experiments were permissive to enhanced intracellular growth even in the presence of IFN-γ.

The ability of the strain MP287/03 to induce in MΦ some properties of the M2 cells, suggested that infection of the MΦ, pretreated with IL-10, by these bacteria may synergize in IL-10- dependent M2 polarization of these cells. The obtained results demonstrated that the treatment with IL-10 led to reduction of the proinflammatory MΦ activation by the studied mycobacterial strains. These cells displayed increased expression of the M2 markers, MR, IL-10 and Arg-1. The highest levels of Arg-1 were observed in the cells infected by MP287/03 mycobacteria, demonstrating that the treatment with IL-10 favored the M2-type activation of these cells.

Although the cells infected with MP287/03 strain displayed increased levels of the M2 markers in the presence or absence of regulating cytokines, these cells secreted high levels of the proinflammatory MIP-2 chemokine. In contrast to the MCP-1 chemokine, regulating monocyte recruitment which is essential for formation of functional granuloma, the continues production of MIP-2, and other chemokines attracting granulocytes, was demonstrated to cause excessive recruitment of neutrophils to the infected lungs, contributing to tissue damage in pulmonary tuberculosis, reviewed by [27]. The high level of MIP-2 secretion and inappropriate proinflammatory MΦ activation, observed in the BMDM cultures infected with MP287/03 strain in this study, may have aggravating implications for *in vivo* infection with these, fast-replicating intracellular bacteria. Verification of this important issue is currently under investigation in our laboratory.

## Conclusions

The data obtained in this study show that the pathogenic Mbv strains differed in their capacity to modulate the M1-type activation phenotype induced by IFN-γ. In contrast to the mycobacterial strains demonstrating moderate ability to grow intracellularly which enhanced classical activation of MΦ by INF-γ, the fast growing strain of Mbv induced an atypical, mixed M1/M2 phenotype, leading to inhibition of MΦ bactericidal activity. These data demonstrate functional diversity of Mbv strains circulating in animal population, highlighting novel strategies of intracellular adaptation of the pathogenic mycobacteria. Elucidating the functional significance of diversity of virulence-associated properties of Mbv is important for understanding the diverse outcomes of infection and mechanisms of pathogenesis of bovine tuberculosis.

## Methods

### Mycobacteria

Two isolates of Mbv from animals with tuberculosis were used in this study. The strain B2 was isolated from buffalo and gently provided by Dr. Eliana Roxo (Biological Institute, USP, São Paulo, Brazil). The bovine strain MP287/03 was kindly provided by Dr. José Soares Ferreira Neto (Institute for Veterinary Medicine, USP, São Paulo, Brazil). *M. tuberculosis* strain H37Rv (ATCC) was kindly provided by Dr. Philip Suffys (Oswaldo Cruz Foundation, FIOCRUZ, Rio de Janeiro, Brazil).

Mycobacterial strains were grown in suspension in complete 7H9 Middlebrook broth (Difco, Detroit, MI), containing 10% albumin dextrose complex, ADC (BD, Sparks, MD), 0.5% glycerol and 0.05% Tween-80 at 37°C under Biosecurity level 3 containment conditions. Additionally, sodium pyruvate 0.4% was added to the cultures of Mbv. Bacterial cultures were grown to mid-logarithmic phase, aliquoted, and stored at −70°C. Before experiments, the aliquots were thawed, resuspended in complete 7H9 medium and cultured for 5 days. Bacterial suspensions were ultrasonicated in water bath to disrupt small clumps and obtain single cell suspensions. The resulted dispersion of bacteria was tested by microscopic examination of the suspension samples stained by the acid-fast staining procedure. The densities of the suspensions were measured by spectrophotometry, and corresponding concentrations were determined by serial dilution plating of each strain on Middlebrook 7H10 agar (Difco, Detroit, MI) plates supplemented with 0.5% glycerol, 10% oleic acid–albumin-dextrose–catalase enrichment, OADC (BD, Sparks, MD), and, additionally, with 0.4% sodium pyruvate in the case of Mbv cultures. After 21 days, total CFU were determined.

### Quantification of mycobacterial growth in 7 H9 broth

The bacterial capacity to grow in 7H9 broth was measured by spectrophotometry. Bacterial suspensions adjusted to OD_600_ = 0.1 were cultured at 37°C for twelve days with daily agitation. Bacterial tubes were then vortexed, ultrasonicated in a water bath, and the OD of suspension was measured. To confirm the lack of significant alteration in OD_600_ readings, colony forming units (CFU) were determined for each culture on day 0 through the plating of appropriate bacterial dilution onto the 7H10 agar.

### Generation of bone marrow- derived macrophages

Bone marrow- derived macrophages (BMDM) were obtained as previously described [28] with some modifications. Briefly, bone marrow cells were flushed from the femur of eight- to ten- week-old specific pathogen-free C57BL/6 mice. The cells were dispersed in Dulbecco’s modified Eagle’s medium, DMEM (Sigma, St Louis, MO), supplemented with 1 mM sodium pyruvate, 2 mM L-glutamine, 0.05 M 2-mercaptoethanol (Gibco BRL, Grand Island, NY), 10% heat-inactivated FBS (Hyclone, Road Logan, UT), 50 μg/ml gentamicin (Gibco BRL, Grand Island, NY), and cultured at 37°C in 5% CO_2_ atmosphere. Nonadherent cells were collected after 18 h, resuspended in the complete DMEM, supplemented with 20% L929 cell-conditioned medium as a source of M-CSF, and cultured for 7 days, replacing the medium on day 3. The monolayer cells were scraped, resuspended in DMEM, supplemented with 2% FBS, without antibiotics, and plated at a concentration of 5 x 10^5^ cell/ml in a 96-well plates, 100 μl/well.

### Treatment with cytokines and infection of cell cultures

The BMDM cultures were incubated overnight, pretreated, or not, with murine recombinant IFN-γ, 100 U/ml (Bioscience, Camarillo, CA), or IL-10, 20 ηg/ml (Bioscience, Camarillo, CA) for 2 h, and infected with single-cell suspensions of mycobacterial strains at MOI 1:1 and 5:1. After 3 h of incubation at 37°C, infected monolayers were washed and incubated for additional 6 d in new aliquots of culture medium. In the pretreated cultures, the cytokines were renewed and were present throughout the incubation period. Cell viability of infected MΦ was monitored by trypan blue exclusion and was over 90% in all experiments.

### Quantification of mycobacterial growth in macrophages

Mycobacterial ability to grow intracellularly was evaluated by colony-forming units (CFU) test in the MΦ cultures infected at a MOI of 1:1. After 0, 3 and 6 d of incubation, cells were lysed with 1% saponin to release intracellular bacteria. Lysates of infected cells were resuspended, transferred into screw caps, vortexed and sonicated in a preheated waterbath sonicator (Unique 800, Brazil) at 37°C for 2 min. Aliquots of the sonicate were diluted 10-fold in PBS, plated in quadriplicates on 7 H10 agar plates and incubated at 37°C for 21 days.

### Cytokine quantification

To study cytokines secreted by infected MΦ, the cell cultures were infected at a MOI 5:1 in the presence or absence of recombinant IFN-γ and IL-10, as indicated above. The infected monolayers were washed and incubated for additional 48 h. After incubation, the culture supernatants were collected, filtered through 0.22 μm Spin-X centrifuge tube filters (Corning, NY), and the supernatant aliquots were stocked at −70°C for posterior cytokine determination. The cells left untreated and cells stimulated with LPS of *Escherichia coli* 011B (Sigma Aldrich, MO), 1 μg/ml, and r-IFN-γ for 48 h were used as a negative and positive control of proinflammatory macrophage activation, respectively. The frozen samples of culture supernatants of the infected BMDM were then thawed and immediately analyzed using Bio-Plex Pro Mouse Cytokine Assay (BioRad Laboratories, Hercules, CA), following the manufacturers protocol. Standard curves for each cytokine were generated using reference cytokine concentrations supplied by the manufacturer.

### Nitric oxide determination

Nitric oxide (NO) generation in the culture supernatants was assessed by the Griess method to measure nitrites, which are stable breakdown products of NO. Briefly, culture supernatant was incubated with the Griess reagents I (1% sulfanilamide in 2.5% phosphoric acid) and II (0.1% naphthylenediamine in 2.5% phosphoric acid). The absorbency was read within 5 min at 550 nm and actual concentration calculated using a standard curve with serial dilutions of sodium nitrite.

### Detection of iNOS, ARG-1 and MR by Western blot

The infected adherent cells were resuspended in lysis buffer (10% SDS, 20% glycerol, 5% 2-mercaptoethanol, 2% bromphenol blue and 1 M Tris HCl, pH 6.8) for western blotting analysis. Cell samples in the lysis buffer were harvested and equal amounts of proteins were electrophoresed in a 10% or 8% sodium SDS-PAGE gel under nonreducing conditions. The proteins were then transferred to nitrocellulose membrane (Amersham Hybond-ECL GE) using standard procedures. After overnight blocking with 0.5% non-fatty milk in PBS, the blots were incubated for 1 hr at room temperature with Ab against iNOS, 1:1000 (Santa Cruz Biotechnology, CA), Arg-1, 1:1000 (BD Bioscience), or MR/CD206, 1:100 (Santa Cruz Biotechnology, CA), dissolved in 0.5% non-fatty milk in PBS. The blots were then washed and incubated with peroxidase-conjugated secondary Ab, 1:8000, for 1 hr at room temperature, and the resulting membranes were developed using diaminobenzidine/H_2_O_2_ as a substrate for peroxidase. Densitometric analysis of the protein bands was performed using the software ImageJ for Windows (NIH, Bethesda, MD). The value for the control condition (untreated cells) was set as 1 and other conditions were recalculated correspondingly to allow ratio comparisons.

### Statistical analysis

Statistical analysis was performed using the unpaired Student’s *t* test, one-way analysis of variance (ANOVA) and Bonferroni procedure for multiple range tests, employing Prism 4 software (GraphPad, San Diego, CA) to assess statistical significance between groups of data defining different error probabilities. A value of p < 0.05 was considered to be significant.

## Abbreviations

AAM, Alternatively Activated Macrophages; Arg-1, Arginase 1; BMDM, Bone Marrow- Derived Macrophages; CAM, Classically Activated Macrophages; IFN-γ, Interferon gamma; IL-10, Interleukin 10; iNOS, Inducible Nitric Oxide Synthase; LPS, Lipopolysaccharide; MΦ, Macrophages; Mbv, Mycobacterium bovis; Mtb, Mycobacterium tuberculosis; MR, Mannose Receptor; MCP-1, Monocyte Chemoattracting Protein; MIP-2, Macrophage Inflammatory Protein 2; NO, Nitric Oxide; ROI, Reactive Oxygen Intermediates; RNI, Reactive Nitrogen Intermediates; TLR, Toll-like Receptors.

## Competing interests

The authors declare that they have no competing interests.

## Authors’ contributions

MRMA performed the experiments and prepared the figures; EPA evaluated growth curves of mycobacteria in MΦ and broth; VL cultured and characterized the mycobacterial strains; TVP established the in vitro model of BMDM infection; EPA, SCMR and FMA carried out the immunoassays; EBL, MRIL and MRMA analyzed the data; EL and MRMA conceived of, designed the study and wrote the manuscript, MREL revised the manuscript critically. All authors read and approved the final manuscript.

## Author details

^1^Laboratory of Biology of Recognition, Universidade Estadual do Norte Fluminense, Campos Rio de Janeiro 28013-602, Brazil. ^2^Department of Immunology, Universidade de São Paulo, São Paulo 05508-900, Brazil.
